# 20-Year Trends in Tobacco Sales and Self-Reported Tobacco Use in the United States, 2000–2020

**DOI:** 10.5888/pcd19.210435

**Published:** 2022-07-28

**Authors:** Lungile Nkosi, Satomi Odani, Israel T. Agaku

**Affiliations:** 1 Sefako Makgatho Health Sciences University, Pretoria, South Africa; 2 Cancer Control Center, Osaka International Cancer Institute, Osaka, Japan; 3 Harvard School of Dental Medicine, Boston, Massachusetts

## Abstract

**Introduction:**

In the past 2 decades, many tobacco control policies were enacted, and several new or modified products were introduced into the US marketplace. Continued tobacco surveillance is critical in this evolving landscape. We examined 20-year trends in tobacco use from sales and self-reported data.

**Methods:**

We obtained data on taxable removals (sales) of cigarettes, cigars, roll-your-own (RYO) tobacco, and pipe tobacco from the US Department of the Treasury. We assessed self-reported past 30-day tobacco use from the National Survey on Drug Use and Health among people aged 18 years or older. Volume sales were standardized to cigarette packs and cigarette pack equivalents (CPEs) and trends measured by using joinpoint and logistic regression.

**Results:**

From 2000 to 2019–2020, declines occurred in per capita sales of cigarettes (101.01 to 42.29 packs/capita), little cigars (0.54 to 0.03 CPEs/capita), and RYO tobacco (1.34 to 0.21 CPEs/capita). Volume sales also decreased for chewing tobacco and scotch/dry snuff (all *P* < .05). Conversely, volume sales increased for pipe tobacco, moist snuff, and snus for the respective assessed periods. Large cigar volume sales did not change significantly. We found consistent trends in self-reported use, except for RYO tobacco (decreased volume sales but increased self-reported use) and pipe smoking (increased volume sales, but trivial self-reported use <1% throughout the study period). Current use of any tobacco product decreased from 32.2% to 22.9% during the assessed period.

**Conclusion:**

Harmonizing the tax and regulatory structure within and across the diversity of tobacco products may help reduce aggregate tobacco consumption in the US.

**Summary**

**What is already known on this topic?**

Aggregate tobacco consumption has declined in recent years, especially for cigarettes.

**What is added by this report?**

Ever and current use of any tobacco product among US adults aged 18 years or older decreased significantly between 2000 and 2020. However, within and across tobacco product types, trends were not consistently downward. While volume sales declined for cigarettes, little cigars, roll-your-own tobacco, chewing tobacco, and scotch/dry snuff, we found increased sales for pipe tobacco, moist snuff, and snus. Volume sales of large cigars did not change significantly.

**What are the implications for public health practice?**

Eliminating imbalances in taxes and regulations between various tobacco product types may help reduce aggregate tobacco consumption.

**MEDSCAPE CME**

In support of improving patient care, this activity has been planned and implemented by Medscape, LLC, and *Preventing Chronic Disease*. Medscape, LLC, is jointly accredited by the Accreditation Council for Continuing Medical Education (ACCME), the Accreditation Council for Pharmacy Education (ACPE), and the American Nurses Credentialing Center (ANCC), to provide continuing education for the healthcare team.

Medscape, LLC, designates this Journal-based CME activity for a maximum of 1.00 AMA PRA Category 1 Credit(s)™. Physicians should claim only the credit commensurate with the extent of their participation in the activity.

Successful completion of this CME activity, which includes participation in the evaluation component, enables the participant to earn up to 1.0 MOC points in the American Board of Internal Medicine’s (ABIM) Maintenance of Certification (MOC) program. Participants will earn MOC points equivalent to the amount of CME credits claimed for the activity. It is the CME activity provider’s responsibility to submit participant completion information to ACCME for the purpose of granting ABIM MOC credit.

Release date: July 28, 2022; Expiration date: July 28, 2023

**Learning Objectives**

Upon completion of this activity, participants will be able to:

1. Evaluate 20-year trends in US tobacco use from 2000 to 2020, overall and for cigarettes and cigars, based on sales and self-reported data
2. Assess 20-year trends in US pipe and roll-your-own tobacco use from 2000 to 2020, based on sales and self-reported data
3. Describe public health implications of 20-year trends in US tobacco use from 2000 to 2020, based on sales and self-reported data

**EDITOR**

Ellen Taratus, MS

Senior Editor

Preventing Chronic Disease

Atlanta, GA

**CME AUTHOR**

Laurie Barclay, MD

Freelance writer and reviewer

Medscape, LLC

Laurie Barclay, MD, has the following relevant financial relationships:

Formerly owned stocks in: AbbVie

**AUTHORS**

Lungile Nkosi, MPH

Sefako Makgatho Health Sciences University, Pretoria, South Africa

Satomi Odani, MPH

Osaka International Cancer Institute, Cancer Control Center, Osaka, Japan

Israel T. Agaku, PhD

Harvard School of Dental Medicine, Boston, MA, United States

## Introduction

Healthy People sets science-based, 10-year national objectives for improving the nation’s health and well-being (1,2). Tobacco-related Healthy People 2030 objectives include reducing adult tobacco use to 16.2% or less (from 20.1% in 2018), current cigarette smoking to 5% or less (from 13.9% in 2019), and any combustible tobacco use (ie, cigarettes, cigars, pipes) to 5% or less (from 16.8% in 2018) (2). Holistically examining long-term trends in the diversity of tobacco products in the US marketplace is important because these products are not independent of one another nor are their trends. Switching from one product to another is largely driven by market forces (eg, product design and marketing) and economic factors (eg, tax imbalances) (3–7). Yet tobacco products that are close substitutes are being regulated differently (8). Little cigars and large cigars are both cigars but are taxed differently, as are pipe and roll-your-own (RYO) tobacco, which are both loose forms of smoking tobacco (5,6). Furthermore, only in August 2016 did the “deeming rule” extend the regulatory authority of the US Food and Drug Administration beyond cigarettes to other similarly harmful combustible products, such as cigars, hookah, and pipe tobacco (9). Yet these newly deemed combustibles are marketed in a plethora of flavors, whereas cigarette flavors are banned (except menthol) (3,10,11).

It is therefore imperative for policy makers to understand the long-term trends in tobacco product consumption and how these trends may have shifted in response to policy interventions (4,6). Such information can help eliminate loopholes that dampen the impact of tobacco control policies through tax avoidance strategies (4–6,12). Some studies have examined trends in US tobacco consumption over varying lengths of time by using both population and sales data (13–15); however, no recent study has examined trends over the past 2 decades to gain insights into longer-term trends that may be different from year-on-year changes, seasonal variations, or even multiyear trends over shorter periods of time. To fill this gap, we examined changes in the consumption of cigarettes, cigars, smokeless tobacco, RYO tobacco, and pipe tobacco during the 20-year period from 2000 to 2020. Analyzed data comprised both volume sales data (2000–2020) and self-reported data (2002–2019).

## Methods

### Data sources

#### Self-reported use of tobacco products

We obtained self-reported data on use of tobacco products from the 2002–2019 National Survey on Drug Use and Health (NSDUH) (16), an annual household-based, nationally representative survey of the US population. Response rates ranged during this period from 61.2% in 2014 to 71.9% in 2002. Data before 2002 were not included in the analysis because NSDUH survey methodology changed in 2002, limiting comparability with previous years; also, 2020 data were not available at the time of analysis. For these reasons, the self-reported data analyzed covered the period 2002–2019. Our analytic sample consisted of adults aged 18 years or older.

We assessed current (≥1 time in the past 30 days) use of cigarettes, cigars (“big cigars, cigarillos, and even little cigars that look like cigarettes”), RYO tobacco, smokeless tobacco (“snuff, dip, chewing tobacco, or ‘snus’”), and pipes. We also assessed ever use (≥1 time during lifetime) for all types of tobacco except RYO tobacco. We defined any tobacco use as use of 1 or more of these products.

#### Tobacco product sales

We obtained data on taxable removals (actual sales) from the US Department of the Treasury for cigarettes, little cigars, large cigars, RYO tobacco, and pipe tobacco during 2000–2020 (17). We obtained data on volume sales for smokeless tobacco products (moist snuff, scotch/dry snuff, chewing tobacco products, and snus) from the US Federal Trade Commission based on filings by the tobacco companies during 2000–2019 (18).

### Analyses

#### Self-reported use of tobacco products

Data were weighted using the survey package in R version 4.0.3 (R Foundation for Statistical Computing) to yield nationally representative estimates. We calculated overall and stratified prevalence estimates. To determine the precision of prevalence estimates, we used relative standard errors (RSEs) calculated by dividing the standard error by the prevalence. Estimates with RSEs of 40% or more were suppressed, consistent with standard practice in which values from 30% to 50% have been used as criteria for suppression (19). We tested linear trends with logistic regression that used orthogonal polynomials adjusted for sex, age, and race and ethnicity.

#### Tobacco product sales

Sales were standardized to packs for cigarettes (20 sticks) and to cigarette pack equivalents (CPEs) for little cigars, pipe tobacco, and RYO tobacco by using standard approaches outlined in previous studies (14,20). The approaches assumed the following: a package of 20 small cigars is equivalent to a pack of 20 cigarettes because it shares the same size, shape, and weight. RYO tobacco CPEs were based on weight (14.6 g tobacco per cigarette pack). Large cigars and smokeless tobacco products were not standardized to CPEs because of the wide variability in composition and size (14). Adult annual per capita sales were estimated by dividing total sales by the number of persons aged 18 years or older (as indicated by the US Census Bureau) (21). We used joinpoint regression to measure trends in estimates of annual percentage change (APC) for subsets of time or segments and average annual percentage change (AAPC) for the entire study period.

## Results

### Cigarette trends

**Figure 1:**
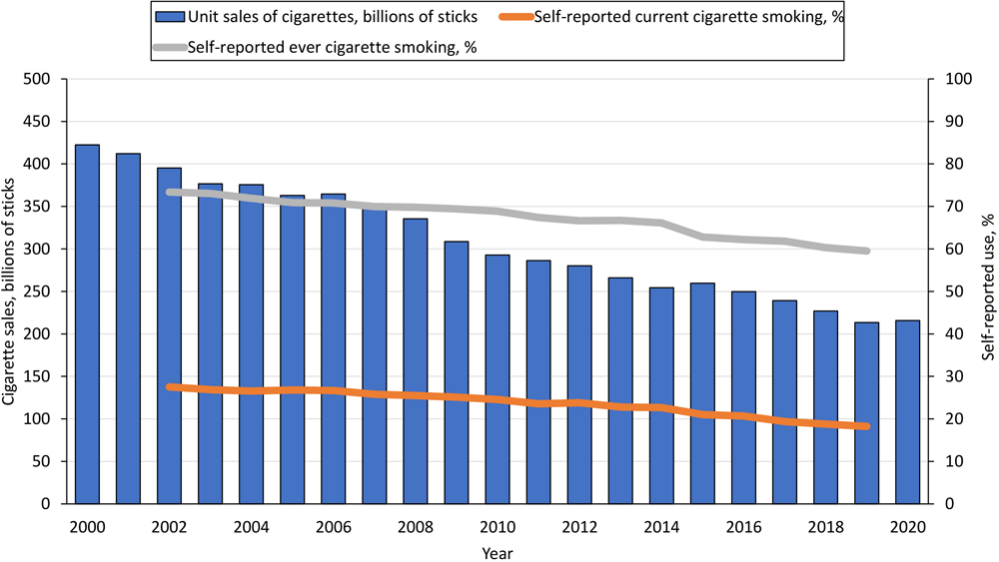
Trends in total sales and self-reported ever and current adult cigarette smoking during 2000–2020, US. Self-reported data on use of tobacco products obtained from the 2002–2019 National Survey on Drug Use and Health (16). Data on sales of cigarettes during 2000–2020 obtained from the US Department of the Treasury (17).

In total, 324.35 billion cigarette packs were sold during the past 20 years combined. Both self-reported data on use and sales data consistently showed steady declines in cigarette consumption (Table 1 and Table 2; Figure 1). Sales declined from 21.12 to 10.79 billion packs during 2000–2020 (AAPC = −3.5%; 95% CI, −3.6% to −3.3%). Expressed as per capita sales, this decline corresponded to a decrease from 101.01 to 42.29 cigarette packs per US adult (Table 1).

**Table 1:** Total Sales of Various Tobacco Products in Standardized Units of Cigarette Packs or CPEs, 2000–2020, US^a^

Year	Cigarettes		Little cigars		Large cigars		Pipe tobacco		Roll-your-own tobacco		Moist snuff	
	Million packs sold^b^	Packs per capita^c^	Million CPEs sold^b^	CPEs per capita	Million sticks sold^d^	Sticks per capita	Million CPEs sold^e^	CPEs per capita	Million CPEs sold^e^	CPEs per capita	Million CPEs sold^f^	CPEs per capita
2000	21,124.76	101.01	112.65	0.54	3,409.69	16.30	169.81	0.81	280.93	1.34	2,049.40	9.80
2001	20,605.07	97.06	108.57	0.51	3,563.75	16.79	152.80	0.72	268.81	1.27	2,129.63	10.03
2002	19,762.18	92.05	112.39	0.52	3,706.29	17.26	143.76	0.67	328.31	1.53	2,205.87	10.27
2003	18,834.13	86.79	114.78	0.53	4,018.50	18.52	129.09	0.59	369.63	1.70	2,298.08	10.59
2004	18,780.37	85.56	135.08	0.62	4,319.16	19.68	121.37	0.55	399.26	1.82	2,435.07	11.09
2005	18,148.6	81.75	188.60	0.85	4,436.11	19.98	120.88	0.54	482.99	2.18	2,522.36	11.36
2006	18,228.49	81.15	209.58	0.93	4,508.08	20.07	107.76	0.48	512.49	2.28	2,581.24	11.49
2007	17,419.64	76.67	238.51	1.05	4,663.03	20.52	97.61	0.43	537.35	2.36	2,741.22	12.06
2008	16,769.2	72.91	273.55	1.19	4,672.74	20.32	98.70	0.43	630.37	2.74	2,800.49	12.18
2009	15,419.39	66.28	107.53	0.46	7,981.90	34.31	333.20	1.43	359.12	1.54	2,919.42	12.55
2010	14,639.62	62.41	44.83	0.19	9,940.95	42.38	691.76	2.95	184.00	0.78	3,086.37	13.16
2011	14,317.63	60.24	37.69	0.16	9,997.56	42.07	1,020.27	4.29	152.22	0.64	3,218.50	13.54
2012	13,991.92	58.25	35.24	0.15	9,438.75	39.30	1,132.15	4.71	131.71	0.55	3,357.06	13.98
2013	13,304.4	54.88	30.53	0.13	7,783.00	32.10	1,212.19	5.00	112.72	0.46	3,482.77	14.37
2014	12,724.33	51.99	27.12	0.11	6,961.14	28.44	1,169.49	4.78	94.53	0.39	3,522.71	14.39
2015	12,986.03	52.57	26.53	0.11	5,757.43	23.31	1,124.42	4.55	107.95	0.44	3,614.78	14.63
2016	12,491.24	50.11	23.77	0.10	5,056.76	20.28	1,077.77	4.32	91.29	0.37	3,716.81	14.91
2017	11,964.83	47.59	20.73	0.08	5,168.61	20.56	1,089.68	4.33	76.51	0.30	3,730.76	14.84
2018	11,345.8	44.78	17.81	0.07	5,018.61	19.81	1,015.58	4.01	60.59	0.24	3,677.15	14.51
2019	10,670.03	41.81	7.75	0.03	4,666.35	18.29	964.12	3.78	56.69	0.22	3,615.30	14.17
2020	10,792.93	42.29	8.70	0.03	4,357.01	17.07	924.51	3.62	53.58	0.21	—	—
AAPC^g^ (95% CI) [*P* value]	−3.5 (−3.6 to −3.3) [<.001]		−12.1 (−19.0 to −4.6) [<.001]		0.7 (−2.3 to 3.9) [.60]		9.4 (7.8 to 11.0) [<.001		−7.7 (−10.7 to −4.6) [<.001]		3.0 (2.7 to 3.4) [<.001]	

Abbreviations: —, does not apply; AAPC, average annual percentage change; CPE, cigarette pack equivalent.

a Data sources: Data on taxable removals (actual sales) of cigarettes, little cigars, large cigars, pipe tobacco, and RYO tobacco were obtained from the Alcohol and Tobacco Tax and Trade Bureau, US Department of the Treasury (17). Data on volume sales of smokeless tobacco were obtained from the US Federal Trade Commission for 2000–2019 (18).

b The number of cigarette packs and CPEs for little cigars was calculated by dividing the number of sticks by 20. Little cigars resemble cigarettes in all respects and so were considered direct equivalents.

c Adult per capita sales based on the US adult population aged ≥18 years using data from the US Census Bureau for each year.

d Large cigars were not converted to CPEs because of variations in size and tobacco content.

e CPEs for pipe and RYO loose smoking tobacco were based on weight (14.6 g tobacco per cigarette pack).

f Moist snuff CPEs assumed the equivalence of a 1.2 oz tin to 2.5 packs of cigarettes based on consumption.

g Overall linear trend during 2000–2020; significant difference defined as *P* < .05.

**Table 2:** Prevalence of Self-Reported Current (Past 30-Day) Use of Cigarettes and Noncigarette Tobacco Products Among US Adults Aged ≥18 Years, by Demographic Characteristics, National Survey on Drug Use and Health, 2002–2019^a^

Characteristic	Cigarettes		Cigars		Roll-your-own tobacco		Pipe		Smokeless tobacco^b^	
	2002	2019	2002	2019	2002	2019	2002	2019	2002	2019
**Overall**	27.4 (26.7–28.1)	18.2 (17.7–18.7)^c^	5.4 (5.1–5.8)	4.6 (4.3–4.8)^c^	2.6 (2.4–2.9)	3.0 (2.8–3.3)^c^	0.8 (0.6–1.0)	0.7 (0.6–0.9)	3.5 (3.2–3.7)	3.3 (3.1–3.5)^c^
**Sex**										
Female	24.5 (23.6–25.5)	16.3 (15.7–17.0)^c^	1.6 (1.4–1.8)	2.0 (1.8–2.3)^c^	1.8 (1.5–2.1)	2.4 (2.1–2.7)^c^	0.3 (0.1–0.4)	0.3 (0.2–0.4)	0.5 (0.3–0.6)	0.6 (0.5–0.7)
Male	30.8 (29.7–31.9)	20.3 (19.5–21.1)^c^	9.6 (9.0–10.3)	7.3 (6.8–7.8)^c^	3.5 (3.1–3.9)	3.7 (3.4–4.1)^c^	1.4 (1.1–1.7)	1.2 (1.0–1.4)	6.7 (6.2–7.3)	6.2 (5.8–6.6)^c^
**Age, y**										
18–25	40.8 (39.9–41.7)	17.8 (17.0–18.6)^c^	11.0 (10.4–11.6)	7.7 (7.1–8.2)^c^	4.5 (4.2–4.9)	3.2 (2.8–3.5)^c^	1.1 (0.9–1.3)	1.3 (1.1–1.5)^c^	4.9 (4.5–5.3)	5.0 (4.5–5.4)^c^
26–34	32.7 (31.1–34.3)	23.6 (22.5–24.8)^c^	6.6 (5.8–7.5)	6.4 (5.8–7.1)	3.4 (2.8–4.1)	3.6 (3.1–4.1)^c^	0.5 (0.3–0.7)	1.0 (0.7–1.3)^c^	5.4 (4.7–6.1)	4.4 (3.8–4.9)
35–49	30.8 (29.6–32.0)	21.6 (20.7–22.6)^c^	5.8 (5.2–6.5)	4.5 (4.1–5.0)^c^	3.2 (2.7–3.7)	3.3 (2.9–3.6)^c^	0.5 (0.3–0.7)	0.6 (0.4–0.7)	3.2 (2.8–3.7)	4.1 (3.7–4.6)^c^
≥50	17.3 (16.0–18.7)	14.7 (13.8–15.5)^c^	2.4 (1.9–2.9)	3.0 (2.6–3.5)	1.0 (0.7–1.4)	2.7 (2.3–3.0)^c^	1.0 (0.6–1.4)	0.6 (0.4–0.8)^c^	2.3 (1.8–2.8)	2.0 (1.7–2.4)
**Race and ethnicity**										
African American	28.2 (26.0–30.4)	20.8 (19.3–22.3)^c^	7.1 (6.0–8.2)	8.7 (7.7–9.6)^c^	4.5 (3.5–5.4)	3.4 (2.8–4.0)	0.4 (0.1–0.7)	0.7 (0.4–0.9)	1.7 (1.1–2.4)	1.3 (0.9–1.8)^c^
Hispanic	25.0 (22.8–27.2)	13.5 (12.4–14.7)^c^	5.2 (4.2–6.3)	3.3 (2.8–3.9)^c^	3.9 (3.0–4.9)	2.2 (1.7–2.6)^c^	0.3 (0.1–0.4)	0.5 (0.3–0.7)^c^	0.5 (0.3–0.8)	0.9 (0.7–1.1)^c^
Other^d^	25.5 (22.1–28.9)	14.5 (12.9–16.1)^c^	2.7 (2.0–3.4)	2.9 (2.3–3.6)	4.2 (2.9–5.5)	2.8 (2.1–3.5)^c^	—e	1.0 (0.6–1.4)	1.8 (1.1–2.4)	2.3 (1.6–2.9)
White	28.0 (27.2–28.8)	19.5 (18.8–20.1)^c^	5.4 (5.0–5.8)	4.3 (4.0–4.7)^c^	2.0 (1.8–2.2)	3.2 (2.9–3.5)^c^	1.0 (0.7–1.2)	0.8 (0.6–0.9)	4.4 (4.0–4.7)	4.5 (4.2–4.8)^c^
**Annual household income, $**										
≤19,999	35.5 (33.8–37.3)	30.1 (28.5–31.6)^c^	5.7 (5.1–6.4)	6.5 (5.8–7.3)^c^	5.4 (4.7–6.2)	7.7 (6.8–8.6)^c^	0.9 (0.7–1.2)	1.5 (1.1–1.9)^c^	3.1 (2.5–3.7)	3.3 (2.7–3.8)
20,000–49,999	29.7 (28.5–30.8)	21.7 (20.7–22.7)^c^	5.0 (4.5–5.5)	4.4 (3.9–4.9)	2.8 (2.4–3.3)	3.6 (3.2–4.1)^c^	0.8 (0.5–1.0)	0.8 (0.6–1.0)	3.7 (3.3–4.2)	3.1 (2.7–3.5)
50,000–74,999	24.6 (23.0–26.3)	17.8 (16.5–19.0)^c^	4.9 (4.1–5.7)	4.2 (3.6–4.8)^c^	1.4 (1.1–1.8)	2.3 (1.8–2.9)^c^	0.8 (0.2–1.4)	0.7 (0.4–0.9)	3.6 (2.9–4.3)	3.4 (2.8–3.9)^c^
≥75,000	19.7 (18.3–21.1)	11.7 (11.1–12.4)^c^	6.3 (5.5–7.1)	4.1 (3.7–4.6)^c^	0.8 (0.6–1.1)	1.2 (1.0–1.4)^c^	0.7 (0.4–1.0)	0.5 (0.3–0.6)	3.2 (2.7–3.8)	3.5 (3.1–3.8)^c^
**Education**										
Less than high school diploma	35.2 (33.3–37.0)	26.7 (25.0–28.4)^c^	5.9 (5.1–6.8)	5.2 (4.4–6.0)	5.2 (4.4–6.0)	6.6 (5.7–7.5)^c^	0.8 (0.5–1.0)	1.0 (0.7–1.3)	4.1 (3.3–4.9)	3.3 (2.7–3.9)
High school diploma	32.3 (31.0–33.6)	25.2 (24.0–26.3)^c^	5.4 (4.8–5.9)	5.3 (4.7–5.9)^c^	3.0 (2.6–3.4)	4.7 (4.2–5.3)^c^	0.8 (0.5–1.1)	1.1 (0.8–1.3)^c^	4.2 (3.7–4.7)	4.5 (4.0–5.0)^c^
Some college	29.0 (27.6–30.5)	20.3 (19.4–21.2)^c^	6.0 (5.3–6.7)	5.0 (4.5–5.5)^c^	2.1 (1.6–2.5)	2.7 (2.3–3.0)^c^	1.0 (0.6–1.5)	0.8 (0.6–1.0)	3.3 (2.8–3.8)	3.9 (3.5–4.3)^c^
College degree or more	14.5 (13.4–15.7)	8.1 (7.5–8.8)^c^	4.6 (4.0–5.3)	3.4 (3.0–3.9)^c^	0.9 (0.6–1.1)	0.8 (0.6–1.0)	0.6 (0.3–0.9)	0.4 (0.2–0.5)	2.3 (1.9–2.8)	1.9 (1.6–2.2)

a Data source: Substance Abuse and Mental Health Services Administration (16). All values are percentage (95% CI).

b Prevalence of smokeless tobacco use showed an increasing trend in the adjusted analysis.

c Significant linear trend during 2002–2019 (*P* < .05). Linear trend assessed in a binary logistic regression model using orthogonal polynomials that adjusted for age, sex, and race and ethnicity.

d Includes American Indian/Alaska Native, Pacific Islander.

e Estimate not presented because relative standard error was ≥40%.

**Table:** 

Year	Unit sales for cigarettes, billions of sticks	Self-reported current cigarette smoking, %	Self-reported ever cigarette smoking, %
2000	422.5	Data not available	Data not available
2001	412.1	Data not available	Data not available
2002	395.2	27.5	73.4
2003	376.7	26.9	73.1
2004	375.6	26.6	71.9
2005	363.0	26.8	70.9
2006	364.6	26.7	70.8
2007	348.4	25.8	70.0
2008	335.4	25.5	69.8
2009	308.7	25.1	69.4
2010	292.8	24.6	68.9
2011	286.4	23.6	67.4
2012	280.1	23.8	66.6
2013	266.1	22.8	66.7
2014	254.5	22.7	66.1
2015	259.7	21.0	62.8
2016	249.8	20.7	62.2
2017	239.3	19.4	61.8
2018	226.9	18.8	60.3
2019	213.4	18.2	59.5
2020	215.9	Data not available	Data not available

Self-reported cigarette smoking decreased significantly during 2002–2019 in the overall population for both ever (73.4% to 59.5%) and current smoking (27.4% to 18.2%) (Table 2 and Table 3). We found the slowest rates of decline in the prevalence of current cigarette smoking by age among adults aged 50 years or older (17.3% to 14.7%; relative percentage change [RPC] = −15.0%); by race or ethnicity, among African American respondents (28.2% to 20.8%; RPC = −26.2%); by annual household income, among those with an income of $19,999 or less (35.5% to 30.1%; RPC = −15.2%); and by education, among those who had less than a high school diploma (35.2% to 26.7%; RPC = −24.1%). These groups also had the highest prevalence of current cigarette smoking in 2019, except for adults aged 50 years or older, who had the lowest prevalence (14.7%). Conversely, we found the largest declines in the prevalence of current cigarette smoking by education among the most educated group (respondents with a college education or more, 14.5% to 8.1%; RPC = −44.1%); by age, among adults aged 18 to 25 years (40.8% to 17.8%; RPC = −56.4%); and by race and ethnicity, among Hispanic respondents (25.0% to 13.5%; RPC = −46.0%).

**Table 3:** Prevalence of Self-Reported Ever Use of Cigarettes and Noncigarette Tobacco Products Among US Adults Aged ≥18 Years, National Survey on Drug Use and Health, 2002–2019^a^

Characteristic	Cigarettes		Cigars		Pipe		Smokeless tobacco	
	2002	2019	2002	2019	2002	2019	2002	2019
Overall	73.4 (72.6–74.2)	59.5 (58.8–60.2)^b^	40.0 (39.1–40.8)	33.8 (33.2–34.5)^b^	18.6 (17.8–19.3)	12.5 (12.0–13.0)^b^	21.2 (20.5–21.9)	16.6 (16.1–17.1)^b^
**Sex**								
Female	68.5 (67.3–69.6)	54.8 (53.9–55.8)^b^	19.3 (18.5–20.2)	18.3 (17.6–19.0)^b^	4.5 (3.9–5.0)	3.8 (3.4–4.2)	6.7 (6.1–7.3)	5.2 (4.9–5.6)^b^
Male	78.7 (77.7–79.7)	64.5 (63.6–65.5)^b^	62.3 (61.1–63.5)	50.5 (49.4–51.5)^b^	33.8 (32.6–35.1)	21.7 (20.9–22.6)^b^	36.9 (35.7–38.0)	28.8 (27.9–29.7)^b^
**Age, y**								
18–25	71.3 (70.5–72.1)	43.4 (42.3–44.4)^b^	45.8 (44.9–46.7)	30.8 (29.8–31.8)^b^	8.1 (7.6–8.6)	7.9 (7.3–8.4)^b^	23.9 (23.2–24.7)	16.6 (15.8–17.4)^b^
26–34	73.0 (71.4–74.6)	60.0 (58.7–61.4)^b^	41.5 (39.8–43.2)	40.1 (38.8–41.5)	9.5 (8.6–10.5)	10.6 (9.7–11.4)^b^	29.1 (27.6–30.6)	20.8 (19.7–21.9)^b^
35–49	75.8 (74.6–77.0)	62.5 (61.3–63.6)^b^	42.2 (40.9–43.6)	37.1 (36.0–38.3)^b^	16.7 (15.7–17.7)	8.6 (8.0–9.3)^b^	21.4 (20.3–22.5)	20.0 (19.1–21.0)^b^
≥50	72.4 (70.8–74.0)	62.5 (61.3–63.7)^b^	35.1 (33.4–36.8)	30.8 (29.6–31.9)^b^	28.2 (26.6–29.9)	16.5 (15.6–17.5)^b^	16.4 (15.1–17.7)	13.3 (12.4–14.1)^b^
**Race/ethnicity**								
African American	63.7 (61.3–66.1)	45.2 (43.3–47.1)^b^	29.8 (27.4–32.2)	24.5 (22.9–26.1)^b^	12.3 (10.3–14.3)	5.5 (4.6–6.3)^b^	13.5 (11.6–15.3)	6.9 (5.9–7.8)^b^
Hispanic	61.1 (58.5–63.7)	46.4 (44.6–48.1)^b^	27.6 (25.4–29.8)	23.1 (21.6–24.5)^b^	6.8 (5.3–8.3)	5.2 (4.4–6.0)^b^	9.0 (7.7–10.4)	8.6 (7.7–9.6)
Other	59.6 (55.4–63.7)	41.8 (39.4–44.2)^b^	26.9 (23.5–30.3)	22.4 (20.4–24.3)^b^	9.9 (7.6–12.3)	7.2 (6.0–8.4)^b^	13.6 (11.2–15.9)	9.7 (8.5–10.9)^b^
White	78.1 (77.2–78.9)	68.1 (67.3–68.9)^b^	44.7 (43.7–45.7)	40.0 (39.1–40.8)^b^	22.2 (21.3–23.1)	16.4 (15.7–17.1)^b^	25.1 (24.2–25.9)	21.4 (20.7–22.1)^b^
**Annual household income, $**								
≤19,999	67.9 (66.1–69.7)	57.1 (55.4–58.8)^b^	30.7 (29.1–32.4)	25.5 (24.0–27.0)^b^	12.9 (11.6–14.2)	11.1 (10.0–12.2)^b^	18.3 (16.8–19.7)	13.7 (12.6–14.9)^b^
20,000–49,999	73.0 (71.8–74.3)	58.9 (57.7–60.2)^b^	37.6 (36.3–38.9)	29.0 (27.8–30.2)^b^	18.1 (17.0–19.2)	11.8 (10.9–12.7)^b^	20.8 (19.8–21.8)	13.7 (12.8–14.5)^b^
50,000–74,999	75.7 (73.9–77.5)	61.2 (59.6–62.9)^b^	43.0 (41.0–45.0)	34.5 (32.8–36.1)^b^	19.2 (17.4–20.9)	12.9 (11.7–14.0)^b^	22.5 (21.0–24.1)	16.6 (15.4–17.8)^b^
≥75,000	76.7 (75.2–78.2)	60.1 (59.0–61.2)^b^	49.1 (47.3–50.9)	40.0 (38.8–41.1)^b^	23.5 (21.9–25.2)	13.3 (12.4–14.1)^b^	23.3 (21.8–24.7)	19.7 (18.8–20.5)
**Education**								
Less than high school diploma	67.1 (65.1–69.1)	54.9 (52.8–56.9)^b^	30.6 (28.7–32.4)	22.3 (20.6–24.0)^b^	14.7 (13.2–16.2)	8.5 (7.4–9.6)^b^	20.5 (18.8–22.3)	12.6 (11.4–13.9)^b^
High school diploma	74.7 (73.3–76.0)	61.5 (60.1–62.8)^b^	37.2 (35.8–38.5)	30.0 (28.7–31.3)^b^	16.6 (15.4–17.8)	11.0 (10.1–11.9)^b^	21.6 (20.5–22.8)	17.4 (16.4–18.4)^b^
Some college	76.7 (75.3–78.1)	62.4 (61.2–63.6)^b^	43.1 (41.4–44.7)	36.0 (34.8–37.1)^b^	18.9 (17.5–20.4)	13.8 (12.9–14.7)^b^	22.0 (20.8–23.3)	18.6 (17.7–19.5)
College degree or more	72.8 (71.2–74.4)	57.1 (55.8–58.4)^b^	47.1 (45.4–48.9)	38.8 (37.6–40.1)^b^	23.5 (21.9–25.0)	13.8 (12.8–14.7)^b^	20.3 (19.0–21.6)	15.5 (14.6–16.4)^b^

a Data source: Substance Abuse and Mental Health Services Administration (16). All values are percentage (95% CI).

b Significant linear trend during 2002–2019 (*P* < .05). Linear trend assessed in a binary logistic regression model using orthogonal polynomials that adjusted for age, sex, and race and ethnicity.

### Little and large cigar trends

**Figure 2:**
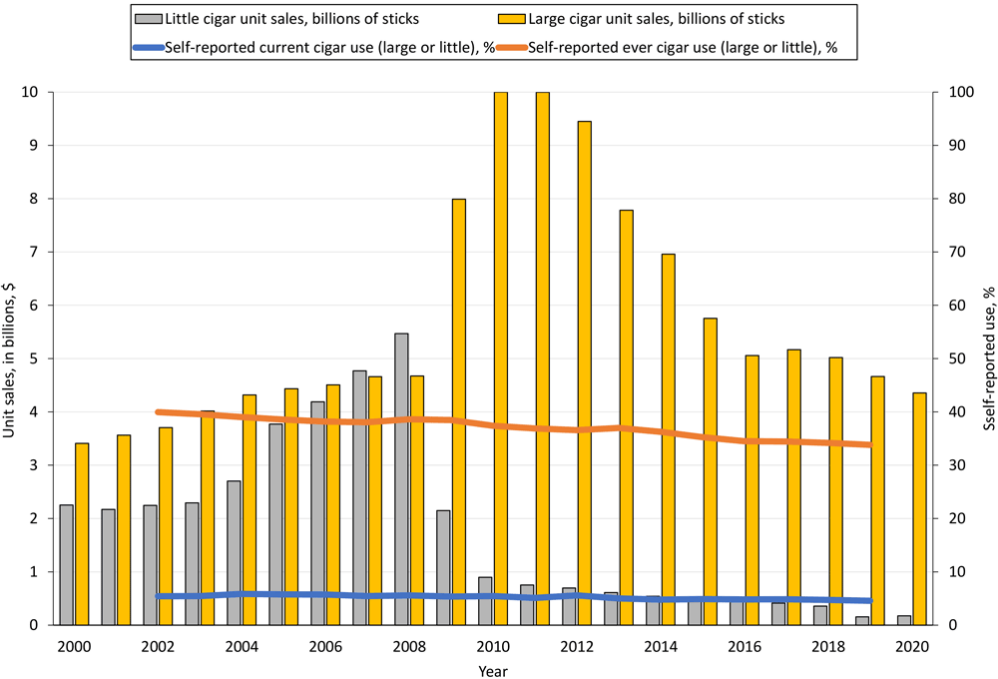
Trends in self-reported current and ever use of cigars (large and little) and cigar unit sales (large and little) during the 20-year period 2000–2020, US. Self-reported data on use of tobacco products obtained from the 2002–2019 National Survey on Drug Use and Health (16). Data on sales of cigars during 2000–2020 obtained from the Department of the Treasury (17).

We found a dramatic shift in volume sales for large cigars after 2009. The volume sales of large cigars exceeded volume sales for little cigars by a factor of only 1.6 before 2007, and even lagged little cigar volume sales in 2007 and 2008. During 2010–2020, 15 large cigars were sold for every 1 little cigar sold. Joinpoint analysis confirmed a rise in volume sales of little cigars from 2000 to 2008 (APC = 12.0%; 95% CI, 6.1%–18.3%), followed by a steep decrease during 2008–2011 (APC = −48.3%; 95% CI, −68.7% to −14.9%), then a brief plateau, and then another steep decline during 2017–2020 (APC = −30.6%; 95% CI, −46.0% to −10.9%). For large cigars, we first found a gradual significant increase during 2000–2007 (APC = 3.6%; 95% CI, 0.7%–6.6%), then a sharp increase during 2007–2010 (APC = 30.9%; 95% CI, 5.9%–61.8%), followed by a significant decrease during 2010–2020 (APC = −8.7%, 95% CI, −10.2% to −7.2%). While the most recent time series segment for large and little cigars showed significant declines, the decline was much steeper for little cigars. Considering the entire 20-year period, no significant change occurred in large cigar volume sales during 2000–2020 overall, but a significant decrease occurred in little cigar volume sales (AAPC = −12.1%; 95% CI, −19.0% to −4.6%) (Table 1, Figure 2).

**Table:** 

Year	Self-reported current cigar use (large or little), %	Self-reported ever cigar use (large or little), %	Little cigar unit sales, billions of sticks	Large cigar unit sales, billions of sticks
2000	—	—	2.3	3.4
2001	—	—	2.2	3.6
2002	5.4	40.0	2.2	3.7
2003	5.5	39.6	2.3	4.0
2004	5.9	39.0	2.7	4.3
2005	5.8	38.6	3.8	4.4
2006	5.8	38.2	4.2	4.5
2007	5.5	38.1	4.8	4.7
2008	5.6	38.6	5.5	4.7
2009	5.4	38.5	2.2	8.0
2010	5.5	37.4	0.9	10.0
2011	5.1	36.9	0.8	10.0
2012	5.6	36.6	0.7	9.5
2013	5.0	37.0	0.6	7.8
2014	4.8	36.9	0.5	7.0
2015	4.9	35.3	0.5	5.8
2016	4.8	34.5	0.5	5.1
2017	4.9	34.4	0.4	5.2
2018	4.7	34.2	0.4	5.0
2019	4.6	33.8	0.2	4.7
2020	—	—	0.2	4.4

A significant decrease during 2002–2019 was also seen in prevalence of self-reported ever (40.0% to 33.8%) and current cigar smoking (5.4% to 4.6%). Analysis of self-reported NSDUH data showed that during 2019, the prevalence of self-reported current cigar smoking was highest by sex among men (7.3%); by age, among adults aged 18 to 25 years (7.7%); by race and ethnicity, among African American respondents (8.7%); by annual household income, among the group with the lowest household income (≤$19,999; 6.5%); and by education, the least educated groups (less than high school diploma, 5.2%; and high school diploma only, 5.3%) (Table 2). While the prevalence of current cigar use decreased among most groups, the prevalence increased among women (1.6% to 2.0%), adults aged 50 years or older (2.4% to 3.0%), and African American respondents (7.1% to 8.7%), making the 2019 prevalence of cigar smoking among African American people 2 times higher than among White people; among the latter, the prevalence declined from 5.4% to 4.3% (all *P* values for trends < .05). The most affluent group (annual household income ≥$75,000) had a striking reduction in the prevalence of current cigar smoking, moving from the group with highest prevalence in 2002 (6.3%) to the lowest prevalence in 2019 (4.1%).

### Pipe and RYO tobacco trends

From perpetually weak sales during 2000–2009, pipe tobacco rose to dominate the loose tobacco market in the post-2009 period (Table 1). The total number of pounds of pipe tobacco sold during 2000–2009 was 47.67 million; this jumped by almost 8 times to 368.84 million pounds during 2010–2020. In contrast, total unit sales for RYO tobacco shrunk to one-third of its historical volume, from 134.20 million pounds during 2000–2009 to 36.17 million pounds during 2010–2020. For every pound of pipe tobacco sold during 2000–2009, an average 3 pounds of RYO tobacco were sold (ranging from a ratio of 1.06 in 2009 to 6.34 in 2008); in contrast, from 2010 onward, for every pound of RYO tobacco sold, an average 12 pounds of pipe tobacco were sold (ranging from a ratio of 3.92 in 2010 to 17.26 in 2020). Pipe tobacco volume sales first rose sharply in 2009 and continued their ascent for the next 4 years (2010, 2011, 2012, 2013), before commencing a gradual decline thereafter. Even with declining pipe tobacco sales, the ratio in total pipe-to-RYO tobacco volume sales continued to increase with each year because although both products declined in sales, the decline for RYO tobacco was steeper. Per capita sales for pipe tobacco increased from 0.81 CPE per capita in 2000 to 5.00 CPE per capita in 2013 and declined to 3.62 in 2020 (Table 1).

Paradoxically, even as the sales of products labeled as pipe tobacco increased dramatically during 2000–2020 (AAPC = 9.4%; 95% CI, 7.8%–11.0%) and unit sales for RYO tobacco decreased (AAPC = −7.7%; 95% CI, −10.7% to −4.6%), self-reported data on use told a completely different story: trends in use of pipe tobacco decreased and trends in use of RYO tobacco increased. The prevalence of ever pipe smoking decreased significantly (18.6% to 12.5%), while the prevalence of current pipe smoking was trivial and remained at 1% or less throughout the study period without any significant change. Conversely, overall prevalence of current RYO tobacco smoking increased significantly from 2.6% to 3.0% during 2002–2019 (all *P* < .05).

We found some segmentation in self-reported NSDUH data in the profiles of respondents who smoked pipe tobacco vs RYO tobacco (Table 2). When we used estimates for ever pipe smoking (as the current use estimates were trivial), we found that the prevalence in 2019 was highest among men (21.7%), adults aged 50 years or older (16.5%), White respondents (16.4%), the group with the highest annual household income (≥$75,000) (13.3%), and the highest educational achievement (college degree or more) (13.8%). Conversely, the prevalence of current RYO tobacco use was highest among the group with the lowest annual household income (<$19,999) (7.7%) — more than 6 times higher than the prevalence among the most affluent group (annual household income of ≥$75,000) (1.2%). Also, the prevalence of current RYO tobacco smoking was approximately 8 times higher among respondents with less than a high school diploma than among respondents with a college degree or more (6.6% vs 0.8%). By age group, adults aged 26 to 34 years had the highest prevalence of current RYO tobacco smoking in 2019 (3.6%), while adults aged 50 years or older had the lowest prevalence (2.7%). Other groups with a relatively high prevalence of current RYO tobacco smoking in 2019 were men (3.7%) and African American respondents (3.4%).

From 2002 to 2019, significant declines in ever pipe smoking occurred among most groups, except those aged 26 to 34 years, among whom a significant increase occurred (9.5% to 10.6%). Some of the largest declines in the prevalence of ever pipe smoking occurred among men (33.8% to 21.7%), adults aged 35 to 49 years (16.7% to 8.6%), African American respondents (12.3% to 5.5%), respondents with an annual household income of $75,000 or more (23.5% to 13.3%), respondents with a college degree or more (23.5% to 13.8%), and respondents with less than a high school diploma (14.7% to 8.5%) (all *P* < .05). For current RYO tobacco smoking, we found increases among both sexes, although the increase was larger among women (1.8% to 2.4%; RPC = 33.3%) than men (3.5% to 3.7%; RPC = 5.7%). By race and ethnicity, White respondents had the lowest prevalence of current RYO tobacco smoking in 2002, but they were the only racial group in which the prevalence increased from 2002 to 2019 (2.0% to 3.2%; RPC = 60.0%). These trends essentially shifted White people from the group with the lowest prevalence of current RYO tobacco smoking in 2002 to a group with a prevalence among the highest in 2019.

### Smokeless tobacco trends

**Figure 3:**
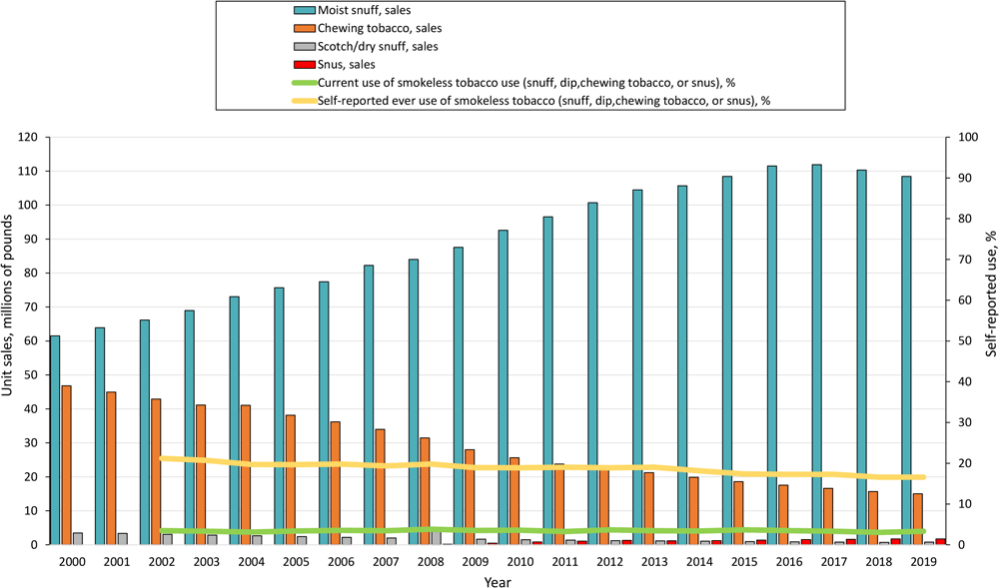
Trends in unit sales for moist snuff, chewing tobacco, scotch/dry snuff, and snus and self-reported current use or ever use of smokeless tobacco (snuff, dip, chewing tobacco, or snus) during the 20-year period 2000–2020, US. Self-reported data on use of tobacco products obtained from the 2002–2019 National Survey on Drug Use and Health (16). Data on sales of smokeless tobacco products obtained from the US Federal Trade Commission for the period 2000–2019 (18).

A significant increase in sales of moist snuff occurred for most of the period 2000–2019, from 61.48 million pounds in 2000 to 108.46 million in 2019 (AAPC = 3.0%; 95% CI, 2.7%–3.4% [Figure 3]). Chewing tobacco sales data during 2000–2019 showed a significant and sustained decrease in sales, from 46.80 million pounds in 2000 to 15.01 million in 2019 (AAPC = −5.9%, 95% CI, −6.2% to −5.5%).

**Table:** 

Year	Unit sales for moist snuff, millions of pounds	Unit sales for chewing tobacco, millions of pounds	Unit sales for scotch/dry snuff, millions of pounds	Unit sales for snus, millions of pounds	Self-reported current smokeless tobacco use (snuff, dip, chewing tobacco, or snus), %	Self-reported ever smokeless tobacco use (snuff, dip, chewing tobacco, or snus), %
2000	61.4	46.8	3.5	0	Data not available	Data not available
2001	63.9	44.9	3.4	0	Data not available	Data not available
2002	66.12	42.9	3.1	0	3.5	21.2
2003	68.9	41.1	2.9	0	3.3	20.7
2004	73.1	41.1	2.7	0	3.1	19.7
2005	75.7	38.1	2.4	0	3.4	19.7
2006	77.4	36.2	2.2	0	3.5	19.8
2007	82.2	34.0	2.0	0	3.4	19.3
2008	84.0	31.4	4.3	0.2	3.8	19.8
2009	87.6	28.0	1.6	0.5	3.5	18.9
2010	92.6	25.6	1.5	0.8	3.6	18.9
2011	96.6	23.8	1.4	1.1	3.2	19.0
2012	100.7	22.3	1.2	1.3	3.7	18.9
2013	104.5	21.2	1.1	1.2	3.4	19.0
2014	105.7	19.9	1.0	1.2	3.4	18.1
2015	108.4	18.6	0.9	1.4	3.7	17.4
2016	111.5	17.5	0.9	1.5	3.5	17.3
2017	111.9	16.6	0.8	1.6	3.37	17.3
2018	110.3	15.7	0.7	1.7	3.0	16.6
2019	108.5	15.0	0.8	1.7	3.3	16.6

Similarly, sales for scotch/dry snuff declined during 2000–2019, from 3.46 million pounds in 2000 to 0.78 million in 2019 (AAPC = −8.6%; 95% CI, −10.1% to −7.1%). The volume sales of snus, for which reporting began in 2008, increased significantly: from 0.17 million pounds in 2008, volume sales increased by more than 10 times, reaching 1.73 million pounds in 2019 (AAPC = 23.3%; 95% CI, 19.7%–26.9%). 

Moist snuff volume sales far outpaced sales of other tobacco products, accounting for 86.1% of all smokeless tobacco volume sales in 2019 (108.5 million pounds of moist snuff sold in that year, of a total of 126 million pounds of any form of smokeless tobacco sold) (Figure 3). Expressed in standardized units, per capita sales (volume) of moist snuff significantly increased from 9.80 CPEs of moist snuff per capita in 2000 to 14.17 in 2019 (*P* < .001). For any form of smokeless tobacco sold, total volume sales in pounds were 111.74 million pounds, which rose for most of the study period to peak in 2016 at 131.43 million pounds, before decreasing to 125.98 million pounds in 2019. The overall trend for the total volume of any smokeless tobacco products sold was upward (AAPC = 0.7; 95% CI, 0.4–0.9).

In 2019, self-reported current smokeless tobacco use by sex was higher among men (6.2%); by age, highest among respondents aged 18 to 25 years (5.0%); by race and ethnicity, highest among White respondents (4.5%); and by education, highest among those with some college education (3.9%) (Table 2). We found significant increases in self-reported current use of smokeless tobacco during 2002–2019 among Hispanic respondents (0.5% to 0.9%), White respondents (4.4% to 4.5%), those aged 18 to 25 years (4.9% to 5.0%) and 35 to 49 years (3.2% to 4.1%), those with household incomes of $75,000 or more (3.2% to 3.5%), those with a high school diploma (4.2% to 4.5%), and those with some college education (3.3% to 3.9%) (all *P* < .05).

### Trends in any tobacco use

Ever use of any tobacco product decreased from 77.3% to 65.9% during 2002–2019, while any current use decreased from 32.2% to 22.9% (all *P* < .05). 

## Discussion

Our results showed a significant decrease in any tobacco use during 2002–2019 in the overall US population. This finding agrees with a recent study that also found declines in any tobacco (cigarettes, cigars, pipe tobacco, and smokeless tobacco) use between 2002 and 2018 (22).

Many segments of the tobacco market shifted in 2009, including the cigar market (becoming heavily dominated by large cigars after 2009) and the loose smoking tobacco market (becoming heavily dominated by pipe tobacco after 2009). Our findings are in line with an official report from the Government Accountability Office that associated these shifts in tobacco sales patterns to a law passed in 2009, the Children’s Health Insurance Program Reauthorization Act (CHIPRA) of 2009, which increased the federal excise tax on cigarettes to $1.01 per pack and equalized the tax rate on RYO tobacco and little cigars to cigarettes (23,24). However, under CHIPRA, larger cigars and other tobacco products were taxed at much lower rates, leading to various tax avoidance behaviors (24,25). As reported previously, many cigar manufacturers switched to or increased production of large cigars thereafter; some even resorted to slightly increasing the weights of their little cigars to be taxed less because the distinction between small and large cigars is based on weight (13,15,24). Similarly, tobacco manufacturers changed the labeling of RYO tobacco after higher taxes were imposed on RYO tobacco than on pipe tobacco; manufacturers began labeling loose tobacco as pipe tobacco to avoid higher taxes and started selling them as “dual use” pipe tobacco to RYO smokers at a lower price (5,6). This might explain our study finding that despite the dramatic increase in pipe tobacco sales and a decrease in RYO tobacco sales, actual self-reported use of RYO tobacco increased while pipe tobacco remained a dying tobacco product with a prevalence of use of less than 1% throughout the study period. Other researchers have reported that pipe tobacco customers were offered cigarette rolling machines after CHIPRA was enacted to help them make affordable cigarettes from RYO-labeled tobacco (26).

Harmonizing regulatory requirements for all tobacco products, including measures such as excise taxes, flavor bans, and minimum pack sizes, may benefit public health. Cigars, for example, are more likely to be sold as single sticks instead of in packs (11,27). The 2009 Family Smoking Prevention and Tobacco Control Act does not include regulations on pack sizes for cigars and cigarillos (28), but these regulations are needed. State and local governments can also act to close these regulatory imbalances within and across different tobacco products, especially considering the wide state variability in how different products are taxed (or not taxed) (3,10). Some jurisdictions prohibited sales of certain flavored tobacco products and raised the minimum tobacco purchase age to 21 years; these actions were also taken by Congress and are now nationwide policy (27,29–31). Outside the US, efforts have also been made to harmonize prices to counter switching of tobacco products as a price-minimizing strategy. For example, the European Union (EU) issued directives to harmonize tobacco taxes and thus reduce the price differences between RYO tobacco and cigarettes; however, studies continued to report an increase in RYO use in EU member states between 2004 and 2015 (32,33). Even though the EU was successful in increasing cigarette and RYO prices between 2004 and 2015, RYO tobacco remained cheaper than cigarettes during that period (32). Taken together, these findings underscore the need for state and local governments to implement tax and nontax measures to close inequalities across tobacco products. Some measures that could be implemented include implementing and enforcing minimum price laws, raising tobacco prices through higher excise taxes, restricting discounting or couponing schemes by tobacco retailers, increasing tobacco retail licensing fees, and implementing disclosure laws for payments or discounts to retailers from tobacco manufacturers.

An element of health equity exists in eliminating imbalances in treatment of cigarette versus noncigarette products because some noncigarette products are disproportionately used among racial and ethnic minority and socioeconomically disadvantaged groups, including cigars among the Black population (11,14,34) and RYO cigarettes among people with low incomes (12,35). The highest prevalence of current cigar use was found among those aged 18 to 25 years, which is a concern because nicotine exposure can harm brain development, which continues well into the third decade of life (36). Besides product regulation, intensified efforts are needed to educate the public about the harms of all forms of tobacco use (37).

### Strengths and limitations

The strengths of our study include the analysis of nationally representative data over 2 decades. This extended duration allowed us to discriminate between long-term trends versus data fluctuations or seasonality, which may be seen in analyses of shorter periods. We also examined trends in products such as snus, chewing tobacco, and moist snuff that are seldom assessed or often collapsed into a single class. Our study synthesized evidence from both self-reported data and taxable removals data (ie, sales), and provides rich insights into the patterns of use and long-term trends.

Nonetheless, some limitations exist. First, other products such as e-cigarettes and hookahs were not included because of a lack of data. Similarly, NSDUH cigar data do not include the type or size of cigar smoked and small and large cigars are classified under “cigars”; therefore, self-reported cigar data from our study do not provide separate estimates for large and small cigars. Second, per capita sales may not accurately measure actual consumption because purchase does not necessarily indicate the use of the products. Third, only taxed tobacco products were included; therefore, per capita data do not include the use of illicit tobacco products. Fourth, CPEs for RYO tobacco may not provide accurate measures because of previously reported price minimizing strategies (ie, putting less tobacco in same-size RYO tobacco packages).

### Conclusion

During the past 20 years, there were upward trends in self-reported use of certain tobacco products (eg, RYO tobacco), downward trends in others (eg, cigarettes and cigars), and no change for some (eg, pipe tobacco). Regulatory imbalances can influence behaviors of both tobacco consumers (switching products) and tobacco manufacturers (shifting products), both reflected in the observed dramatic shifts in substitute products with unequal tax. Public health authorities should remove these loopholes by equalizing taxes across all products, and imposing consistent regulations on flavors, packaging, and other key attributes that increase the accessibility, affordability, availability, and appeal of tobacco products.

## Post-Test Information

To obtain credit, you should first read the journal article. After reading the article, you should be able to answer the following, related, multiple-choice questions. To complete the questions (with a minimum 75% passing score) and earn continuing medical education (CME) credit, please go to http://www.medscape.org/journal/pcd. Credit cannot be obtained for tests completed on paper, although you may use the worksheet below to keep a record of your answers.

You must be a registered user on http://www.medscape.org. If you are not registered on http://www.medscape.org, please click on the “Register” link on the right hand side of the website.

Only one answer is correct for each question. Once you successfully answer all post-test questions, you will be able to view and/or print your certificate. For questions regarding this activity, contact the accredited provider, CME@medscape.net. For technical assistance, contact CME@medscape.net. American Medical Association’s Physician’s Recognition Award (AMA PRA) credits are accepted in the US as evidence of participation in CME activities. For further information on this award, please go to https://www.ama-assn.org. The AMA has determined that physicians not licensed in the US who participate in this CME activity are eligible for AMA PRA Category 1 Credits™. Through agreements that the AMA has made with agencies in some countries, AMA PRA credit may be acceptable as evidence of participation in CME activities. If you are not licensed in the US, please complete the questions online, print the AMA PRA CME credit certificate, and present it to your national medical association for review.

## Post-Test Questions

### Study Title: 20-Year Trends in Tobacco Sales and Self-Reported Tobacco Use in the United States, 2000–2020

#### CME Questions

1. You are advising a physicians’ advocacy group about the likely efficacy of various tobacco control policies. On the basis of the analysis of sales and self-reported data by Nkosi and colleagues, which one of the following statements about 20-year trends in US tobacco use from 2000 to 2019/2020, overall and for cigarettes and cigars, is correct?
  - Among adults aged at least 18 years, ever use of any tobacco product fell during 2002 to 2019, as did any current use
  - Self-reported and sales data showed no significant changes in cigarette consumption
  - Large cigar smoking decreased significantly and consistently, whereas small cigar smoking remained unchanged
  - In 2019, prevalence of self-reported current cigar smoking was highest among White males with the highest household income
2. According to the analysis of sales and self-reported data by Nkosi and colleagues, which one of the following statements about 20-year trends in US pipe and roll-your-own tobacco use from 2000 to 2019/2020 is correct?
  - Pipe tobacco volume sales decreased
  - Per capita sales of roll-your-own tobacco increased
  - Volume sales decreased for moist snuff
  - Per capita sales of chewing tobacco decreased
3. On the basis of the analysis of sales and self-reported data by Nkosi and colleagues, which one of the following statements about public health implications of 20-year trends in US tobacco use from 2000 to 2019/2020 is correct?
  - No factors contributing to major shifts in the US tobacco market from 2009 onward were identified
  - Eliminating imbalances in tax structure and regulations between cigarettes and alternative tobacco products may help reduce US aggregate tobacco consumption
  - Price changes were not likely to affect personal preferences for tobacco products and therefore are not likely to affect overall trends in sales
  - Public health authorities should selectively increase taxes on the most dangerous products
